# Creation of Metal-Complex-Integrated Tensegrity Triangle DNA Crystals

**DOI:** 10.3390/molecules29194674

**Published:** 2024-10-01

**Authors:** Katsuhiko Abe, Haruhiko Eki, Yuki Hirose, Soyoung Park, Shanmugavel Chinnathambi, Ganesh Pandian Namasivayam, Kazuki Takeda, Hiroshi Sugiyama, Masayuki Endo

**Affiliations:** 1 Department of Chemistry, Graduate School of Science, Kyoto University, Sakyo-ku, Kyoto 606-8502, Kyoto, Japan; 2 Department Institute for Integrated Cell-Material Sciences (WPI-iCeMS), Kyoto University, Sakyo-ku, Kyoto 606-8501, Kyoto, Japan; 3 Immunology Frontier Research Center (IFReC), Osaka University, Suita 565-0871, Osaka, Japan; 4 Research Development Division, Kansai University, Suita 565-8680, Osaka, Japan

**Keywords:** tensegrity triangle DNA, DNA crystal, metal complex, DNA nanotechnology, X-ray crystallography

## Abstract

Structural DNA nanotechnology is an emerging field and is expected to be used for various applications in materials science. In this study, we designed a DNA tensegrity triangle to accommodate the bipyridine complexes with metal ions (Ni^2+^ and Fe^2+^) at the center of the space within the triangle. A metal–bipyridine-incorporated DNA tensegrity triangle was crystalized, and the presence of metals within it was confirmed through X-ray crystal structure analysis. A signal of the anomalous dispersion effect derived from metal was observed in the center of the DNA triangle.

## 1. Introduction

The field of nanotechnology is rapidly expanding, and nanometer-scale structures are being constructed from various biomolecules such as nucleic acids [1,2,3], peptide [4], and lipids [5,6]. These biomolecule-based nanostructures are used to create functional nanomaterials and nanodevices [1,2,3,4] and used for biological applications such as drug delivery [5,6]. Nanostructures made of nucleic acids, especially DNA, are attracting attention due to their ability to be designed based on accurate base pairing system for formation of specific shapes and sizes [1,2,3]. In recent years, complex DNA structures such as DNA origami [7,8,9,10,11] and DNA nanomachines [12,13] have been reported. Despite the use of these molecules, it remains unattainable to construct millimeter-scale DNA nanostructures applicable for the functional materials.

**Figure 1:**
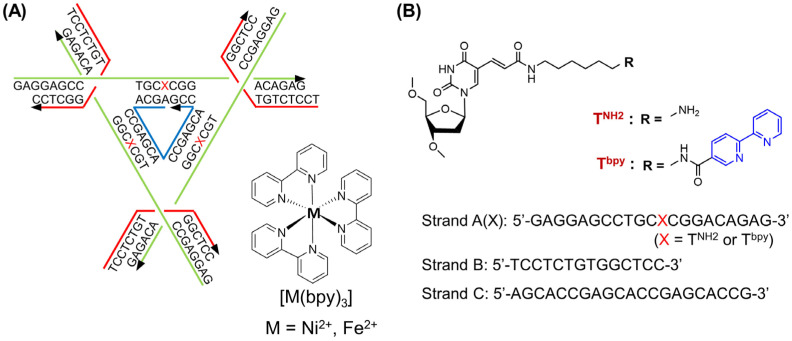
Conceptual diagram of tensegrity triangle structure and the modification: (**A**) A schematic illustration of the tensegrity triangle and bipyridine–metal complex [M(bpy)_3_]. Arrows (5’ to 3’) show strand A (green), strand B (red) and strand C (blue). X indicates modified T with amino (T^NH2^) and bipyridyl (T^bpy^) group. (**B**) Chemical structures of the T^NH2^ and T^bpy^ incorporated to the strand A(X) [A(T^NH2^) and A(T^bpy^)] and their sequences.

In the field of materials science, porous materials such as metal–organic frameworks (MOFs) have been developed, which have designed nanospace for functionalization used for reaction catalysis, gas storage, molecule and ion separation, and drug delivery [14]. DNA crystals also have a nanometer-scale reaction space for the possible incorporation of various molecules inside like porous materials [15,16,17,18]. The tensegrity triangle is a three-fold rotational symmetric DNA motif, consisting of three DNA duplexes arranged in a regular triangle shape [15,16,17,18]. The three double strands are connected by three four-arm branch junctions and have six sticky ends (Figure 1). This sticky end complements the sticky ends of other triangles, allowing for the connection and formation of a 3D periodic lattice or crystal ranging in size from hundreds of µm to mm. Using this structure, it was possible to examine how crystallization susceptibility changed through altering the type and length of the sticky end bases [19]. To construct crystals with a strong system against heat, physical force, and low-concentration salt, DNA strands were covalently connected to each other at the sticky ends using DNA ligase [20]. We previously reported the observation of the real-time assembly of the tensegrity triangle in growing crystals using fast-scanning AFM [21]. In addition, DNA tensegrity triangle crystals allow for the incorporation of functional molecules such as fluorescence dyes [22,23] and redox active materials [24]. From these studies, the tensegrity triangle structure has been utilized to accumulate guest molecules into target positions in the DNA crystals for further application. 

Metal complexes exhibiting electrochemical and photochemical properties are incorporated into the DNA strands to express unique functions [25,26]. Ta three-way branched dsDNA structure was used for the introduction of metal complex with three bpy ligands into the center of the junction [27]. Therefore, the nanometer-scale space consisting of three DNA helices can be used to coordinate the metal ion with three ligands for metal complex formation. 

In this study, we designed a DNA tensegrity triangle to accommodate the metal–bipyridine complex, [Ni(bpy)_3_]^2+^ and [Fe(bpy)_3_]^2+^, in the center space of the triangle (Figure 1) [21]. The formation of the metal complex in the tensegrity triangle was confirmed via UV/vis spectroscopy measurement, and the stability was examined via melting temperature measurement. The crystal structure and position of the metal ion in the crystal were verified using X-ray crystal structure analysis. We also discussed the validity of structural analysis using in silico molecular modeling.

## 2. Results and Discussion

### 2.1. Formation of the Metal Complex in the DNA Tensegrity Triangle

**Figure 2:**
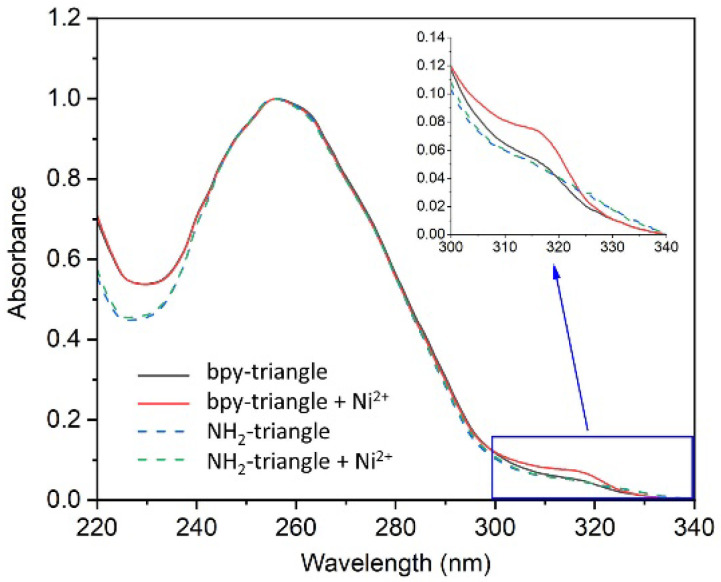
UV/vis spectra of tensegrity triangle with/without bipyridine and Ni^2+^ ion in solution. Inset: Expansion of spectra for the absorption of the bpy ligand part. Spectral change was observed in the solution with the bipyridine triangle in the presence of Ni^2+^ ion (black and red lines).

As a guest molecule, we introduced a nickel–bipyridine complex [Ni(bpy)_3_]^2+^ into the center of the triangular space. We incorporated the bpy ligand into the DNA strand with appropriate linker length to form a rigid complex with metal ions (Figure 1). First, the bipyridine derivative was incorporated to the amino linker at the 5-position of thymine base in the DNA strand (A(T^NH2^)) using bipyridine *N*-hydroxysuccinimide ester (Scheme S1) [28]. After the reaction, the produced strand A(T^bpy^) was purified by HPLC (Figure S1), verified using mass spectrometry, and characterized via UV-vis spectra (Figure S2). The tensegrity triangle structure was assembled from three DNA strands according to the previous studies [27]. DNA strands A(T^bpy^), B, and C and Ni^2+^ ion were annealed to form the tensegrity triangle with nickel–bipyridine complex. Formation of the metal complex [Ni(bpy)_3_]^2+^ was confirmed via measurement of the UV-Vis spectra of the solution. When the tensegrity triangle solution contains strand A(T ^bpy^) with Ni^2+^ ion (10 equivalent), a noticeable spectral change at the 315 nm was observed (Figure 2). However, the spectral change was not observed when using the tensegrity triangle with A(T ^NH2^) in the presence of Ni^2+^ ion. The results indicate that this change is caused by the metal complex formation between the bpy ligands in the strand A(T^bpy^) and Ni^2+^ ion. The spectral change observed is consistent with that of a previous study [24], indicating that the nickel–bipyridine complex was formed in the tensegrity triangle in solution. The stability of the bpy-tensegrity triangle in the presence and absence of Ni^2+^ ion was examined via melting temperature (*T*_m_) measurement (Figure S3). The *T*_m_ of the bpy tensegrity triangle in the absence and presence of Ni^2+^ ion was 39.0 °C and 42.1 °C, respectively, indicating that the formation of the nickel–bipyridine complex clearly stabilized the structure. From these results, the position of the bpy ligand connected to the linker at the 5-position of the thymine base is sufficient for the immobilization of the [Ni(bpy)_3_]^2+^ complex in the tensegrity triangle structure. 

### 2.2. Crystallization of the Metal-Complex-Incorporated DNA Tensegrity Triangle

**Figure 3:**
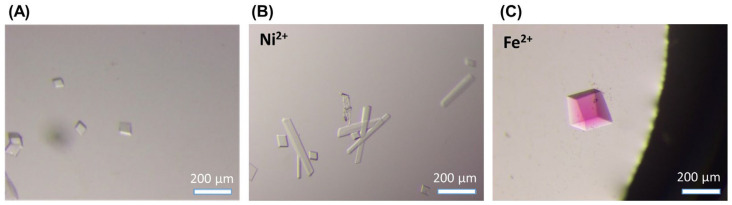
Crystallization of tensegrity triangle with Ni^2+^- or Fe^2+^–bipyridine complex. Microscope images of the DNA crystals: (**A**) the crystals of the tensegrity triangle without the metal ion; (**B**) the crystals of the tensegrity triangle with the Ni^2+^-bpy complex; (**C**) the crystals of the tensegrity triangle with the Fe^2+^-bpy complex.

We next prepared a tensegrity triangle crystal with the metal–bipyridine complex. We used Ni^2+^ and Fe^2+^ ions for complex formation. The procedure to form the DNA crystals followed that reported previously [21]. First, we used three strands, A(T^bpy^), B, and C, without metal ion for crystallization. After annealing, crystallization was performed via a sitting drop vapor diffusion method. Cubic crystals around 50 µm in length appeared within several days, indicating that the bpy ligand modification in strand A did not prevent the crystallization (Figure 3A). Next, DNA crystals with the nickel–bipyridine complex or iron–bipyridine complex were prepared. Using the same procedure in the presence of Ni^2+^ and Fe^2+^, crystals containing nickel–bipyridine and iron–bipyridine complexes formed within a day, and sufficiently large crystals (around 50 µm in length) were obtained after several days (Figure 3B,C). In the presence of the Ni^2+^ ion, the crystal formed cubic and rectangular shapes (Figure 3B). When the Fe^2+^ ion was introduced, red-colored cubic crystals caused by Fe^2+^ ion chelation to bpy ligands were obtained (Figure 3C). These DNA crystals with the metal complex were not grown into larger size—as compared to the crystals using unmodified DNA strands (up to 500 µm) [21]—probably because of the modification and some structural stress caused by metal complex formation in the tensegrity triangle crystal. Although the crystal size is small, we successfully obtained enough sizes of the DNA crystals for the next X-ray crystal structure analysis. 

### 2.3. Analysis of the DNA Tensegrity Triangle Crystals with Metal Complexes

To determine the chelation of the metal ions in the tensegrity triangle crystals, we conducted an X-ray irradiation experiment for the structural analysis of the crystal with the Ni^2+^-bpy and the Fe^2+^-bpy complexes. The diffraction data of the DNA crystal containing iron–bipyridine complex were collected using the beamline BL41XU of SPring-8 (Harima, Japan). Diffraction measurement was performed using X-rays with a wavelength of 1.74 Å. As a result of the analysis, the same space group *H*3, as in the previous study, was obtained. In the case of the Ni^2+^-bpy complex in the tensegrity triangle crystal, the resolution obtained was as low as 6.56 Å; however, the structure could be determined via the molecular replacement method. The electron density map corresponding to the lattice of the tensegrity triangle (blue mesh) and metal (green mech) in the middle of the triangle was confirmed (Figure S4). From this electron density map, atomic details of the structure of the Ni^2+^-bpy complex could not be determined. 

**Figure 4:**
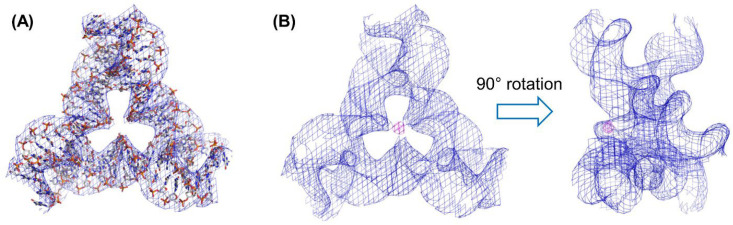
The 2*F*_o_-*F*_c_ electron density maps of Fe^2+^-bpy tensegrity triangle and anomalous dispersion effects: (**A**) the 2*F*_0_-*F*_c_ map (blue mesh) obtained via the molecular replacement method using the initial structure (PDB ID: 3GBI) is shown at 1σ level; (**B**) the superimposition of the 2*F*_o_-*F*_c_ map (1σ) (blue mesh) and the anomalous difference Fourier map (3σ) (magenta mesh) are shown.

Next, we carried out X-ray diffraction measurement of tensegrity triangle crystal with Fe^2+^-bpy complex. Analysis of the diffraction data of Fe^2+^-bpy complex in the tensegrity triangle crystal was performed. The resolution using Fe^2+^ ion was 4.93 Å, which was better than that using the Ni^2+^ ion because the anomalous dispersion effect of iron is large. The phase could be determined via the molecular replacement method (Figure 4A). The electron density map corresponding to the lattice of the tensegrity triangle (blue mesh) was confirmed; however, the atomic details of the structure of the Fe^2+^-bpy complex could not be determined. Due to the anomalous dispersion of iron, a signal corresponding to iron was observed at the center of the triangle with 6.1σ intensity (magenta mesh) (Figure 4B). From the side view, a blob of electron density of iron protruding slightly from the triangle was observed (Figure 4B right). These results show that the metal complexes have been successfully incorporated at the expected location in the tensegrity triangle crystals. 

### 2.4. Modeling of the Metal Complex in the Tensegrity Triangle Based on the Crystal Structure

**Figure 5:**
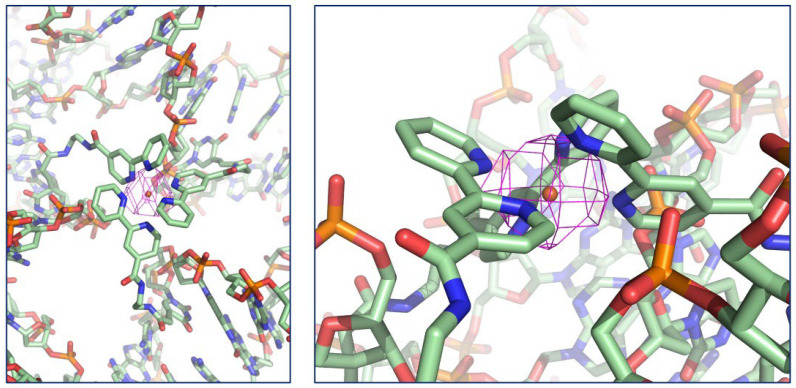
The model structure of tensegrity triangle crystal with the [Fe(bpy)_3_]^2+^ complex based on in silico molecular modeling and a map of the anomalous dispersion effect at the 3σ level (purple mesh). Fe^2+^ ion is located in the center of three bpy ligands and purple mesh.

In this X-ray crystal structure analysis, it was not possible to obtain data with sufficient resolution to determine the metal complex structure. However, as mentioned above, a strong signal due to iron’s anomalous dispersion effect was observed in the anomalous difference Fourier map. Therefore, by assuming the structure of the metal complex part from existing structural data, we conducted in silico molecular modeling. In the modeling, the positions of the DNA and iron were fixed. The model structure is shown in Figure 5. In the model, bipyridine ligands in the tensegrity triangle crystal are spatially arranged in reasonable positions. This indicates that the length of the linker from the 5-position of the thymine base in strand A are suitable to position spatially and arrange the bipyridine ligands for coordination of the Fe^2+^ ion. From these modeling results, we confirmed that the [Fe(bpy)_3_]^2+^ complex formed in the crystal spatially occupies the center of the tensegrity triangle in a reasonable manner, as designed.

In this study, we have successfully incorporated bipyridine ligands in the tensegrity triangle and precisely positioned the metal complex in the crystal space via self-assembly. It was confirmed that the target molecules were arranged as designed based on spectrum measurements and the X-ray crystallographic analysis. This results demonstrate that the transition metal complex can be incorporated into the target positions using the crystallization of the tensegrity triangle DNA. In the future, it will be possible to produce functional crystal materials that can catalyze reactions such as photochemical and redox-active systems, mimicking heme and cytochrome C by introducing the desired functional molecules. Also, catalytic nucleic acids such as DNA and RNA aptamers can be incorporated inside the DNA crystals to process biochemical reactions and sense small molecule analytes. By introducing stimuli-responsive molecules into the crystal, such as the molecule-responsive and photo-responsive system, it will be possible to utilize them for storing and releasing the desired molecules intensively. This DNA crystal system is expected to serve to create functional biomaterials for various applications in nanoscience, materials science, and medical science.

## 3. Materials and Methods

### 3.1. Material

Unmodified DNA strands were purchased from Eurofins Genomics (Tokyo, Japan), and modified DNA strands were purchased from Japan Bio Service (Saitama, Japan). DNA sequences are as follows:

Strand A(X): 5′-GAGGAGCCTGCXCGGACAGAG-3′ (X = T^NH2^ or T^bpy^);
Strand B: 5′-TCCTCTGTGGCTCC-3′;
Strand C: 5′-AGCACCGAGCACCGAGCACCG-3′.

### 3.2. Synthesis, Purification, and the Identification of Strand A(T^bpy^)

A total of 100 µL of 25% DMF/water (*v*/*v*) solution containing 200 µM DNA strand A(T^NH2^), 50 mM sodium carbonate buffer (pH 9.0), and 1 mM 5-carboxybipyridine *N*-hydroxy succinimide ester^1^ was kept at 40 °C for 3 h (Scheme S1) [28]. The reaction mixture was passed through a spin column, and the product was purified via reversed-phase HPLC (JASCO) using a linear gradient of 2–40% acetonitrile (25min) with 20 mM ammonium formate. (Figure S1). The UV/vis spectra of the A(T^NH2^) and A(T^bpy^) were obtained by NanoDrop One (ThermoFisher, Waltham, MA, USA) (Figure S2A). The attachment of bpy was identified by MALDI-TOF MS (Bruker, Billerica, MA, USA) (Figure S2B). Analytical MALDI-TOF MS: *m*/*z* calcd. for A(T^bpy^) [M+H]^+^: 6857, found; 6862. 

### 3.3. Assembly of DNA Triangle and Measurement of UV Spectra

DNA strands A(X) (X = T^NH2^ and T^bpy^), B, and C were mixed at a molar ratio of 3:3:1 in the buffer consisting of 500 mM lithium sulfate, 25 mM sodium cacodylate (pH 6.0), 125 mM magnesium acetate, and 1.5mM lithium chloride (or without lithium chloride). DNA concentration was 5 µM in total. This solution was annealed from 60 °C to 20 °C in 1 h. After annealing, the UV/vis spectrum of each solution was measured by NanoDrop One (ThermoFisher).

### 3.4. Crystallization of DNA via Sitting Drop Vapor Diffusion Method

DNA strands A(X) (X = T^NH2^ and T^bpy^), B, and C were mixed at a molar ratio of 3:3:1 in the buffer, same as above, and 1 mM nickel(Ⅱ) chloride or 1 mM iron(Ⅱ) chloride was added. DNA concentration was 5 µM, and the drop amount was 10 µL in total. After annealing, the drops were incubated against 0.5 mL of 1.7 M ammonium sulfate aqueous solution at 20 °C for several days using the sitting drop vapor diffusion method. 

### 3.5. Data Collection and Crystal Structure Analysis

The diffraction data of the DNA crystal containing iron–bipyridine complex were collected using X-rays with a wavelength of 1.74 Å at the beamline BL41XU of SPring-8 (Harima, Japan). Diffraction images were integrated with the XDS program [29]. The crystallographic statistics are listed in Table S1. The phase was determined by the molecular replacement method using the reported structure data (PDB ID: 3GBI) by Phenix [30]. Iron in the DNA crystal was detected on the anomalous difference Fourier map. The *R*_work_ and *R*_free_ values after rigid-body and group B-factor refinement were 12.97% and 14.95%, respectively. Creating figures was conducted with PyMOL.

### 3.6. In Silico Molecular Modeling of Fe–Bipyridine Complex

The molecular modeling study was performed with Discovery Studio (BIOVIA) using a CHARMm force field. The initial structure was built based on the crystal structure. The bond orders of several bonds in the linker and bipyridine moieties were manually modified, and finally, the structure of these moieties was modified to obtain a reasonable structure using the clean geometry function. The structure was solvated in cubic water with 10 mM magnesium chloride. Throughout the energy minimization calculations, fixed constraints were applied to the Fe^2+^ ion and the DNA triangle, except for the linkers, the bipyridine moieties, and the modified dTMPs. The structure was pre-minimized with distance restraints between the nitrogen atoms at bipyridine rings and the Fe^2+^ ion. The structure was finally minimized to the stage where the RMS was less than 0.001 kcal/mol·Å via a conjugate gradient algorithm with no restraint at the linkers, the bipyridine moieties, and the modified dTMPs.

## Data Availability

Detailed data is available from the authors.

## Supplementary Materials

The following supporting information can be downloaded at https://www.mdpi.com/article/10.3390/molecules29194674/s1: Scheme S1: Synthesis of DNA strand A(T^bpy^); Figure S1: HPLC profiles for the DNA strand A(TX) before (left) and after (right) the reaction with bpy-succinimide ester; Figure S2: Identification of bpy-modified DNA strand A(T^bpy^); Table S1: Data collection and structure refinement statics.

## References

[1] Seeman N.C. (2010). Nanomaterials based on DNA. Annu. Rev. Biochem..

[2] Hong F., Zhang F., Liu Y., Yan H. (2017). DNA origami: Scaffolds for creating higher order structures. Chem. Rev..

[3] Ge Z., Gu H., Li Q., Fan C. (2018). Concept and development of framework nucleic acids. J. Am. Chem. Soc..

[4] Kuan S.L., Bergamini F.R.G., Weil T. (2018). Functional protein nanostructures: A chemical toolbox. Chem. Soc. Rev..

[5] Hou X., Zaks T., Langer R., Dong Y. (2021). Lipid nanoparticles for mRNA delivery. Nat. Rev. Mater..

[6] Assefi M., Ataeinaeini M., Nazari A., Gholipour A., Vertiz-Osores J.J., Calla-Vásquez K.M., Al-Naqeeb B.Z.T., Jassim K.H., Kalajahi H.G., Yasamineh S. (2023). A state-of-the-art review on solid lipid nanoparticles as a nanovaccines delivery system. J. Drug. Deliv. Sci. Technol..

[7] Rothemund P.W.K. (2006). Folding DNA to create nanoscale shapes and patterns. Nature.

[8] Zhang F., Nangreave J., Liu Y., Yan H. (2014). Structural DNA nanotechnology: State of the art and future perspective. J. Am. Chem. Soc..

[9] Endo M., Sugiyama H. (2014). Single-molecule imaging of dynamic motions of biomolecules in DNA origami nanostructures using high-speed atomic force microscopy. Acc. Chem. Res..

[10] Krissanaprasit A., Key C.M., Pontula S., Labean T.H. (2021). Self-assembling nucleic acid nanostructures functionalized with aptamers. Chem. Rev..

[11] Rossi-Gendron C., El Fakih F., Bourdon L., Nakazawa K., Finkel J., Triomphe N., Chocron L., Endo M., Sugiyama H., Bellot G. (2023). Isothermal self-assembly of multicomponent and evolutive DNA nanostructures. Nat. Nanotechnol..

[12] Douglas S.M., Bachelet I., Church G.M. (2012). A logic-gated nanorobot for targeted transport of molecular payloads. Science.

[13] Li S., Jiang Q., Liu S., Zhang Y., Tian Y., Song C., Wang J., Zou Y., Jerson G., Han J.Y. (2018). A DNA nanorobot functions as a cancer therapeutic in response to a molecular trigger in vivo. Nat. Biotechnol..

[14] Wang Q., Astruc D. (2020). State of the art and prospects in metal–organic framework (MOF)-based and MOF-derived nanocatalysis. Chem. Rev..

[15] Liu D., Wang M., Deng Z., Walulu R., Mao C. (2004). Tensegrity: Construction of rigid DNA triangles with flexible four-arm DNA junctions. J. Am. Chem. Soc..

[16] Zheng J., Birktoft J.J., Chen Y., Wang T., Sha R., Constantinou P.E., Ginell S.L., Mao C., Seeman N.C. (2009). From molecular to macroscopic via the rational design of a self-assembled 3D DNA crystal. Nature.

[17] Lu B., Vecchioni S., Ohayon Y.P., Sha R., Woloszyn K., Yang B., Mao C., Seeman N.C. (2021). 3D hexagonal arrangement of DNA tensegrity triangles. ACS Nano.

[18] Lu B., Woloszyn K., Ohayon Y.P., Yang B., Zhang C., Mao C., Seeman N.C., Vecchioni S., Sha R. (2023). Programmable 3D Hexagonal Geometry of DNA Tensegrity Triangles. Angew. Chem. Int. Ed..

[19] Ohayon Y.P., Hernandez C., Chandrasekaran A.R., Wang X., Abdallah H.O., Jong M.A., Mohsen M.G., Sha R., Birktoft J.J., Lukeman P.S. (2019). Designing higher resolution self-assembled 3D DNA crystals via strand terminus modifications. ACS Nano.

[20] Li Z., Liu L., Zheng M., Zhao J., Seeman N.C., Mao C. (2019). Making engineered 3D DNA crystals robust. J. Am. Chem. Soc..

[21] Eki H., Abe K., Sugiyama H., Endo M. (2021). Nanoscopic observation of a DNA crystal surface and its dynamic formation and degradation using atomic force microscopy. Chem. Commun..

[22] Hao Y., Kristiansen M., Sha R., Birktoft J.J., Hernandez C., Mao C., Seeman N.C. (2017). A device that operates within a self-assembled 3D DNA crystal. Nat. Chem..

[23] Stahl E., Praetorius F., de Oliveira Mann C.C., Hopfner K.P., Dietz H. (2016). Impact of heterogeneity and lattice bond strength on DNA triangle crystal growth. ACS Nano.

[24] Wang X., Sha R., Kristiansen M., Hernandez C., Hao Y., Mao C., Canary J.W., Seeman N.C. (2017). An Organic Semiconductor Organized into 3D DNA Arrays by “Bottom-up” Rational Design. Angew. Chem. Int. Ed..

[25] Stulz E. (2017). Nanoarchitectonics with porphyrin functionalized DNA. Acc. Chem. Res..

[26] Vecchioni S., Lu B., Livernois W., Ohayon Y.P., Yoder J.B., Yang C., Woloszyn K., Bernfeld W., Anantram M.P., Canary J.W. (2023). Metal-Mediated DNA Nanotechnology in 3D: Structural Library by Templated Diffraction. Adv. Mater..

[27] Duprey J.-L.H.A., Takezawa Y., Shionoya M. (2013). Metal-locked DNA three-way junction. Angew. Chem. Int. Ed..

[28] Lin J., Qin B., Fang Z. (2019). Nickel Bipyridine (Ni(bpy)3Cl2) Complex Used as Molecular Catalyst for Photocatalytic CO_2_ Reduction. Catal. Lett..

[29] Kabsch W. (2010). xds. Acta Crystallogr. Sect. D Biol. Crystallogr..

[30] Afonine P.V., Grosse-Kunstleve R.W., Echols N., Headd J.J., Moriarty N.W., Mustyakimov M., Terwilliger T.C., Urzhumtsev A., Zwart P.H., Adams P.D. (2012). Towards automated crystallographic structure refinement with phenix. Refine. Acta Crystallogr. Sect. D Biol. Crystallogr..

