# Supplementary material for: Creation of Metal-Complex-Integrated Tensegrity Triangle DNA Crystals
Source: Molecules. 2024 Oct 1;29(19):4674. doi: 10.3390/molecules29194674 (PMC11478291; doi:10.3390/molecules29194674)
Supplement: Supplementary file 1 [file molecules-29-04674-s001.zip › molecules-3171814-supplementary.pdf]

Supplementary Materials

Creation of Metal-Complex-Integrated Tensegrity Triangle DNA Crystals

Katsuhiko Abe [1], Haruhiko Eki [1], Yuki Hirose [1], Soyoung Park [2,3], Shanmugavel Chinnathambi [2], Ganesh Pandian Namasivayam [2], Kazuki Takeda [1], Hiroshi Sugiyama [2] and Masayuki Endo [2,4,*]

[1] Department of Chemistry, Graduate School of Science, Kyoto University, Sakyo-ku, Kyoto 606-8502, Kyoto, Japan
[2] Department Institute for Integrated Cell-Material Sciences (WPI-iCeMS), Kyoto University, Sakyo-ku, Kyoto 606-8501, Kyoto, Japan
[3] Immunology Frontier Research Center (IFReC), Osaka University, Suita 565-0871, Osaka, Japan
[4] Research Development Division, Kansai University, Suita 565-8680, Osaka, Japan
* Correspondence: endo@kansai-u.ac.jp; Tel.: +81-6-6368-1111

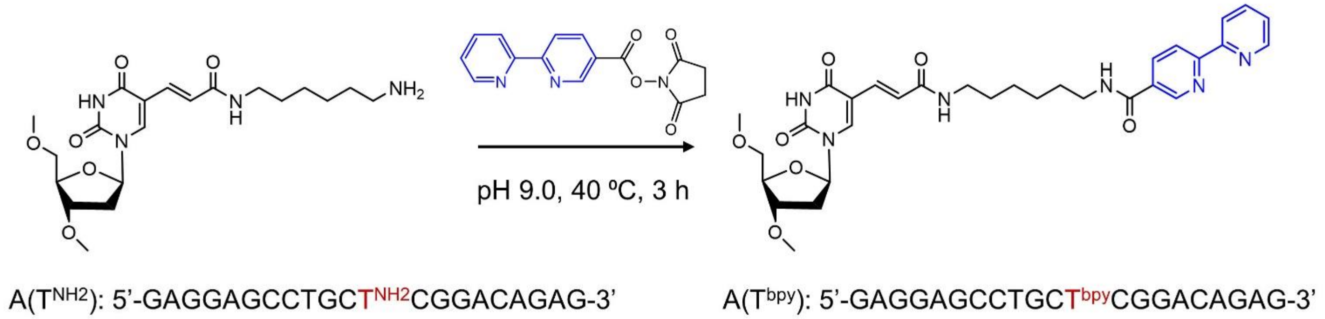

Scheme S1 Synthesis of DNA strand A(T^{bpy}).

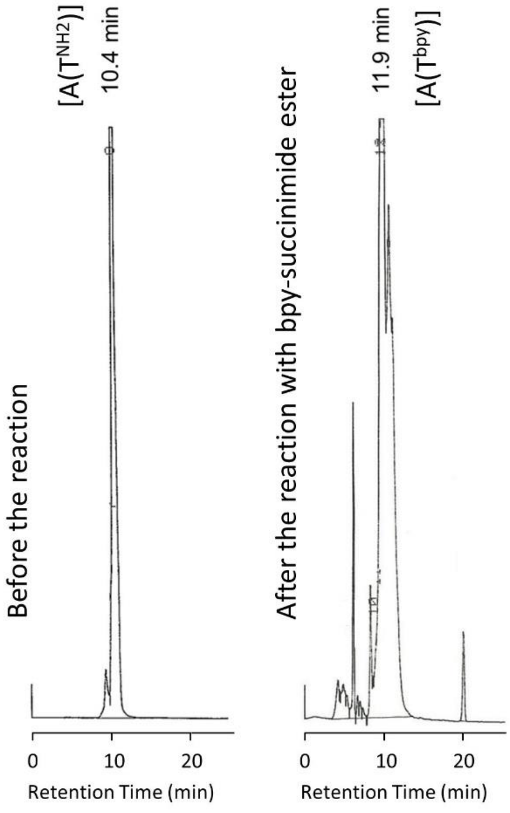

Figure S1. HPLC profiles for the DNA strand A(T^{X}) before (left) and after (right) the reaction with bpy-succinimide ester. After the reaction, the fraction of the main peak was collected.

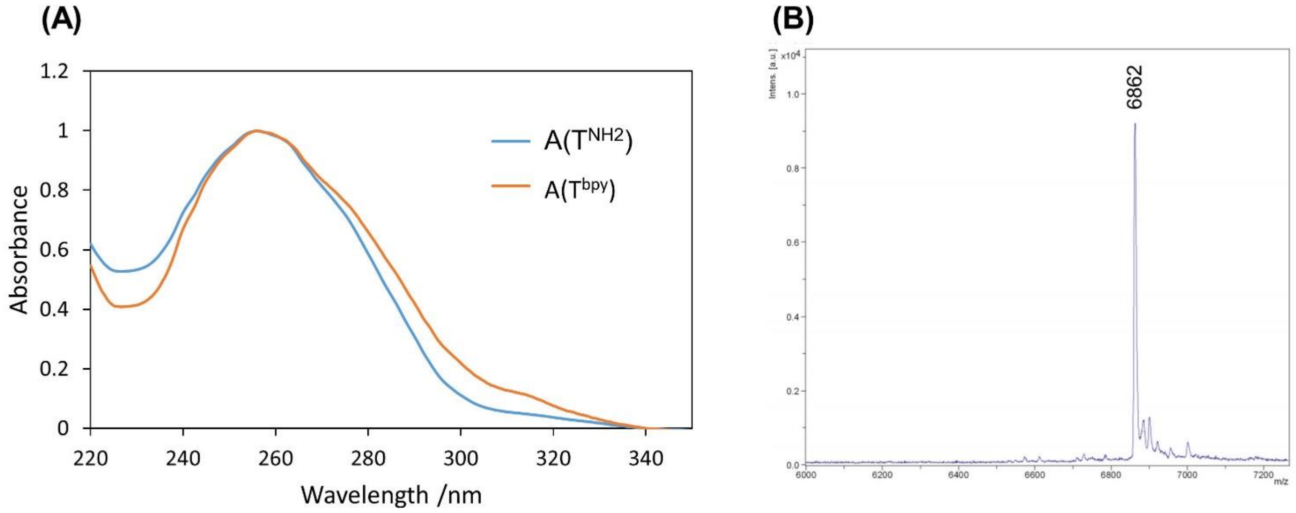

Figure S2. Identification of bpy-modified DNA strand A(T^{bpy}). (A) UV/Vis spectra of DNA strand A(T^{NH2}) and A(T^{bpy}). (B) MALDI-TOF MS profile for HPLC-purified DNA strand A(T^{bpy}). Main peak observed at m/z 6862.

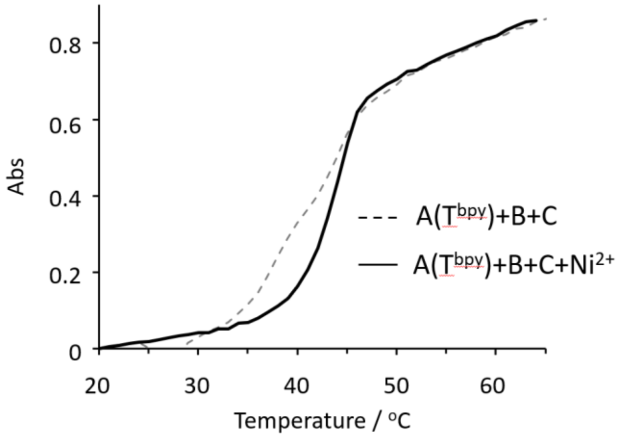

Figure S3. Meting temperature (T_m) measurement of tensegrity triangle [strand A(T^{bpy}) + B + C] in the presence and absence of Ni^{2+} ion. Strand concentration 0.02 μg/μL, 10 mM Tris-HCl (pH 7.6), +0.5 °C/min. T_m (A(T^{bpy})+B+C) 39.0 °C, T_m (A(T^{bpy})+B+C+Ni^{2+}) 42.1 °C

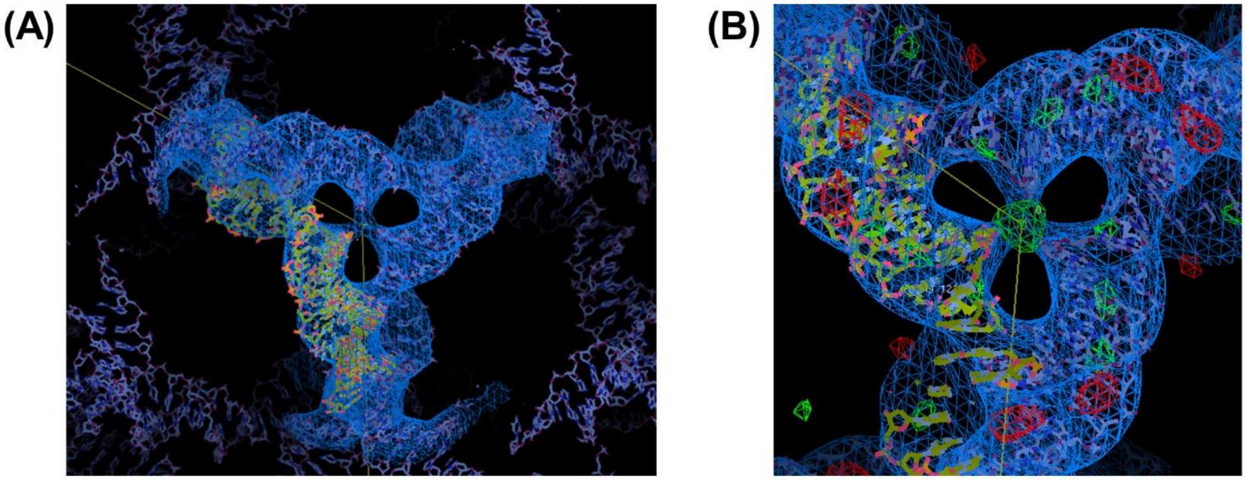

Figure S4. Electron density maps of 2Fo-Fc and superimposition of the DNA tensegrity triangle with Ni-bpy complex. (A) 2Fo-Fc electron density map (blue mesh) obtained by molecular replacement method using the initial structure (PDB ID: 3GBI) is shown at 1σ level. (B) A close-up view of (A). The Fo-Fc map is superimposed on the 2Fo-Fc map. The green mesh is the Fo-Fc map contoured at +3σ showing the excess electron density not included in the initial model, and the red mesh is the map at -3σ.

Table S1. Data collection and structure refinement statics of tensegrity triangle crystal with $[Fe(bpy)_3]^{2+}$.

		Refined data
Diffraction Data		
	Space group	*H*3
	Cell dimensions	
	a, *b*, *c*, Å	107.53, 107.53, 89.63
	α, β, γ, °	90, 90, 120
	Wavelength, Å	1.7400
	Resolution, Å	50-4.93 (5.23-4.93)
	R_{merge}*, %	5.7 (230.9)
	$<I/\sigma(I)>$*	12.13 (0.61)
	$CC_{1/2}$*, %	99.8 (66.5)
	Completeness*, %	99.8 (99.6)
	No. of unique reflections†	1741 (276)
	Redundancy	10.3 (10.2)
Refinement		
	Resolution, Å	41.23-5.89
	R_{work} / R_{free} , %‡	12.97 / 14.95
	No. of atoms	855
	B factors, $Å^2$	359.1

*Values for the highest-resolution shell are in parentheses. †Bijvoet pairs were kept separate. ‡R_{free} was calculated using 5% of the reflections that were not included in the refinement as a test set.
